# Cervus and cucumis peptides ameliorates bone erosion in experimental arthritis by inhibiting osteoclastogenesis

**DOI:** 10.1136/lupus-2019-000331

**Published:** 2019-05-11

**Authors:** Ze-Min Lin, Yu-Ting Liu, Yan-Sheng Xu, Xiao-Qian Yang, Feng-Hua Zhu, Wei Tang, Shi-Jun He, Jian-Ping Zuo

**Affiliations:** 1 Laboratory of Immunopharmacology, State Key Laboratory of Drug Research, Shanghai Institute of Materia Medica Chinese Academy of Sciences, Shanghai, China; 2 University of Chinese Academy of Sciences, Beijing, China; 3 Laboratory of Immunology and Virology, Shanghai University of Traditional Chinese Medicine, Shanghai, China

**Keywords:** cervus and cucumis peptides, rheumatoid arthritis, adjuvant-induced arthritis, collagen-induced arthritis, osteoclastogenesis

## Abstract

**Objective:**

Rheumatoid arthritis is an autoimmune disease characterised by inflammation and bone loss, leading to joint destruction and deformity. The cervus and cucumis polypeptide (CCP) injection, one of the traditional Chinese medicine injections combined extracts from deer horn and sweet melon seeds, is widely used to treat arthritis and bone fracture in China. The present study investigated the therapeutic efficacy and mechanism of CCP on pathological immune cells and bone homoeostasis in rodent experimental arthritis.

**Methods:**

The effects of CCP (4 mg/kg and 2 mg/kg) on clinical arthritis symptoms, bone erosion, proinflammatory cytokines and pathological immune cells induced by complete Freund’s adjuvant was evaluated in male Sprague-Dawley rats. The impacts of CCP (2 mg/kg) on joint erythema and swelling, production of pathogenic antibodies and the proportion of inflammatory cells were assessed in collagen-induced arthritis (CIA) in DBA/1J mice. Regulation of osteoclastogenesis by CCP was observed in the murine macrophage-like RAW264.7 cells treated with receptor activator of nuclear factor-κB ligand (RANKL) and macrophage colony-stimulating factor (M-CSF).

**Results:**

CCP administration significantly prevented disease progression in both adjuvant-induced arthritis (AIA) rats and CIA mice. The therapeutic benefits were accompanied by reduction of paw oedema, reversed bone destruction, decreased pathological changes and osteoclast numbers in joints in AIA rats, as well as attenuated clinical manifestation and autoantibodies production in CIA mice. Meanwhile, in vitro supplemented of CCP concentration dependently inhibited RANKL/M-CSF-induced osteoclast differentiation, without showing cytotoxicity in RAW264.7 cells. Further, the presence of CCP dampened the augmented downstream signalling transduction as well as activation of osteoclast-specific genes and transcription factors induced by RANKL/M-CSF in RAW264.7 cells.

**Conclusion:**

Our study suggested that the therapeutic effects of CCP in experimental arthritis could be attributed to its intervention on RANKL-induced osteoclastogenesis signalling pathway in osteoclast precursor cells.

## Introduction

Rheumatoid arthritis (RA) is a chronic, systemic inflammatory disorder that affects the joints, connective tissues, muscle, tendons and fibrous tissue.^1^

It is becoming increasingly clear that bone destruction in RA is mainly attributable to the abnormal differentiation of osteoclasts.^2^ Proinflammatory cytokines and systemic hormones were frequently reported to be osteoclastogenic.^3^ In particular, macrophage colony-stimulating factor (M-CSF) and receptor activator of nuclear factor-κB ligand (RANKL), the triggers of osteoclast differentiation, could promote invasion of the periosteal surface adjacent to articular cartilage in RA progression.^1^ The subsequent ubiquitination of tartrate resistant acid phosphatase 6 (TRAP6) promotes the association between TGF-β-activated kinase 1 (TAK1) and its binding protein (TAB), thus initiates phosphorylation of the mitogen-activated protein kinases kinase (MAPKKs), that further phosphorylate the MAPKs, including p38 and c-Jun N-terminal kinase.^4 5^

Among the MAPKKs, MKK3 and MKK6 act as activators of p38 during osteoclastogenesis. Osteoclasts and their precursors express only the p38α protein, the major regulator of nuclear factor for activated T cells 1 (NFATc1, encoding a master transcription factor regulating osteoclastogenesis) expression.^6^ Absence of MKK3^7^ or MKK6^8^ in primary bone marrow cells conditioned with M-CSF and RANKL resulted in reduced p38 phosphorylation, decreased expressions of NFATc1 and other osteoclast marker gene regulated by NFATc1. Besides, overexpression of the dendritic cell-specific transmembrane protein (DC-STAMP) in transgenic mice resulted in a phenotype with accelerated cell-to-cell fusion during osteoclast precursor differentiation.^9^

The cervus and cucumis polypeptide (CCP) injection, one of the traditional Chinese medicine injections combined extracts from deer (*Cervus nippon Temminck*) horn and sweet melon (*Cucumis melo L*.) seeds, is widely used in orthopaedics and rehabilitation in recent years.^10^ Here, we reported the effect of CCP on bone destruction in the adjuvant-induced arthritis (AIA) rats and collagen-induced arthritis (CIA) mice, both of which are classic experimental model of arthritis.^11–13^ Furthermore, we demonstrated that CCP exerts direct inhibitory effects on RANKL/M-CSF-induced osteoclast differentiation in the murine macrophagic cell line RAW264.7, by impeding the activation of critical cellular responses during osteoclastogenesis. As we known, systemic lupus erythematosus (SLE) is a prototype of an autoimmune syndrome that is characterised by various clusters of organ manifestations, and arthritis and arthralgia have been noted in up to 95% of patients with SLE.^14 15^ Thus, CCP injection might be a complementary therapy for patients with SLE with lupus arthritis.

## Materials and methods

### Animals and ethics committee

Male Sprague-Dawley (SD) rats and male DBA/1J mice were purchased from Shanghai Laboratory Animal Center of the Chinese Academy of Sciences. All rats and mice were housed in individually ventilated cages with free access to standard laboratory water and food, and kept in a 12 hours light/dark cycle with controlled humidity and temperature.

### Drugs and preparation

The CCP injection (Songmeile injection) was obtained from Harbin Gloria Pharmaceuticals, Harbin, China (Batch number: 150916). Methotrexate (MTX) injection was purchased from Shaanxi Pude Pharmaceutical, Shaanxi, China (Batch number: 036.60901).

The CCP injection is manufactured according to the standard issued by Chines Pharmacopoeia Commission (Number:WS1-XG-002-2002-2005). Briefly: The peptide extracted from the limb bones of healthy adult *Cervus nippon Temminck* and from the dry matured seeds of *Cucumis melo L*. in China, are mixed at the ratio of 2:1 in volume. After necessary concentration, the total peptide content in final solution is more than 3 mg/mL. The endotoxin in the CCP injection is less than 4 EU/mL. As described by Wang *et al*^16^, the average content of amino acids in CCP extract was (in μg/mL): Glu 34.61, Ala 32.38, Asp 30.75, Arg 18.47, Gly 17.14, Val 11.93, Leu 10.12, Tyr 9.74, Phe 9.09, Ile 8.31, Ser 8.1, Pro 7.92, Thr 5.41, Cys 4.23, Met 2.84, and His 2.64. The total content of free amino acids was between 200 and 400μg/mL.

### Induction of experimental arthritis and treatment

The male SD rats were randomly divided into five groups (n≥6 per group, named Control, Saline, MTX, CCP 2 mg/kg and CCP 4 mg/kg), four groups were injected at the right hind metatarsal footpad with 0.1 mL complete Freund’s adjuvant (CFA) contained 10 mg/mL heat-killed *Mycobacterium tuberculosis* H37Ra (Difco, BD Biosciences, Franklin Lakes, New Jersey, USA).^17^ Control rats were injected with 0.1 mL saline. From day 0 of CFA injection, the rats were administered intraperitoneally with saline, MTX (1 mg/kg/day) or CCP (4 mg/kg/day or 2 mg/kg/day) for consecutive 29 days, while the control groups were administered with equal volume of sterilised saline. The arthritis severity and the body weight of rats were monitored two times a week.

The male DBA/1J mice were randomly divided into four groups (n≥6 per group, named Control, Saline, MTX and CCP), three groups were immunised and boosted by bovine type II collagen (CII, Tokyo, Japan) emulsion to induce CIA as previously described,^18^ and the control group received CFA and incomplete Freund’s adjuvant without collagen, respectively. From day 10 after booster immunisation, immunised groups were treated intraperitoneally with saline, MTX (1 mg/kg/day) or CCP (2 mg/kg/day), respectively, for consecutive 56 days, while controls were administered with saline.

### Clinical assessment of arthritis

Body weight and the arthritis score were measured/calculated at the indicated time for both AIA rats and CIA mice. The clinical severity of arthritis was scored as previously described.^19^ The volume of left hind paws was measured with an electronic water plethysmograph from the day of injection (basic value, day 0) and then repeated measurement on day 5, 8, 12, 15, 19, 22, 26 and 29.

The volume of paw swelling was calculated as previously described^20^:

Oedema volume=V_t_ V0, where V_0_ is the volume before CFA injection (ml); V_t_ is the volume at t day after CFA injection (mL).

### Micro-CT analyses

CT images of left hind paws and ankle joints of the rats in all three groups were acquired at the end of treatment, using a Micro-CT scanner as previously described.^18^

### Histological examination and TRAP staining

Rats were sacrificed, and their secondary hind limbs were isolated and fixed in 10% formalin, then decalcified, embedded in paraffin and sectioned. The sections (3 µm) of ankle joints were stained with H&E, and were examined microscopically (magnification 50×).^21 22^ Inflammation, pannus formation, cellular infiltration, synovial proliferation and cartilage erosion were scored on a scale of 0–3 (0: absent; 1: weak; 2: moderate; 3: severe).^22^

To analyse osteoclast in joint tissues, each joint section was treated sequentially with a commercial acid phosphatase leucocyte kit (Sigma-Aldrich, St. Louis, Missouri, USA) to detect the TRAP enzyme. The osteoclasts in field of whole section (magnification 200×) were counted.

### Cytokine and antibodies assays

Cytokines in serum were detected using rat IL-1β, IL-6, IL-17A and TNF-α (BD Biosciences, San Diego, California, USA) ELISA kits according to the manufacturer’s instructions. Levels of CII specific antibodies in serum were measured by ELISA as previously described.^18^

### Flow cytometric analysis

The percentage of Th17 or Th1 cells in spleen was conducted and analysed as our previously reported methods.^18^

### Cell viability

Mouse macrophage RAW264.7 cell line was obtained from the American Type Culture Collection. To evaluate the cytotoxicity of CCP on RAW264.7 cells, cell viability assays were performed using the Cell Counting Kit-8 (CCK-8) as previously reported.^23 24^ Briefly, RAW264.7 cells were seeded in 96-well plates at a density of 2×10^3^ cells/well. After overnight incubation at 37℃, medium was replaced with 100 µL of fresh media containing CCP of various concentrations for 4 days, respectively. Then, CCK-8 solution was added to each well followed by further 2-hour incubation at 37℃. Absorbance was measured at 450 nm with a microplate reader (Spectramax 190, Molecular Devices Corporation, Sunnyvale, California, USA).

### In vitro osteoclastogenesis assay

For differentiation of osteoclasts, RAW264.7 cells were cultured with 10% FBS DMEM containing 50 ng/mL of recombinant mouse RANKL (Sigma-Aldrich), 40 ng/mL recombinant mouse M-CSF (R&D, Minneapolis, Minnesota, USA) in the absence or presence of CCP at indicated concentrations for 4 days.^25 26^

TRAP staining was performed with the aid of a commercial kit (Sigma-Aldrich) according to the manufacturer’s instructions. TRAP-positive multinucleated cells containing three or more nuclei were counted.

### Western blotting

RAW264.7 cells were conditioned with RANKL (50 ng/mL) and M-CSF (30 ng/mL), in presence or absence of CPP for 72 hours.

Equal amounts of protein were separated on 10% SDS-PAGE and western blotted with antibodies to TAK1(D94D), phosphorylated-TAK1(Ser412), MKK6(D31D1) phosphorylated-MKK3(Ser189)/MKK6(Ser206) (Cell Signaling Technology, Beverly, Massachusetts, USA) and α-tubulin (Abcam, Cambridge, UK). Signals were detected using an ECL Plus Western Blotting Detection Reagents (GE Healthcare, Piscataway, New Jersey, USA) according to the manufacturer’s instructions.

### Gene expression analysis

The messenger RNA (mRNA) expressions of the osteoclastogenesis-related genes and transcriptional factors were assessed using RT-PCR assay as previously described.^24^ Briefly, RAW264.7 cells were seeded in 6-well plates (2×10^6^ cells/well), pretreated with various concentrations of CCP (100 µg/mL, 10 µg/mL, 1 µg/mL) for 2 hours, and then were conditioned with mouse recombinant RANKL (50 ng/mL) for further 24 hours. The primers used for PCR amplification are listed in online supplementary table S1.

10.1136/lupus-2019-000331.supp1 Supplementary data

### Statistical analysis

Statistical analysis was performed using GraphPad Prism V.6.0 statistical software. For experiment involving multiple groups, one-way analysis of variance (ANOVA) followed by Turkey’s multiple comparison test was used, except for analysis of arthritis scores and oedema volume which used two-way ANOVA followed by Dunnett’s multiple comparison test. P values less than 0.05 were considered significant.

## Results

### CCP treatment prevented arthritis progression in rodent arthritis

**Figure 1:**
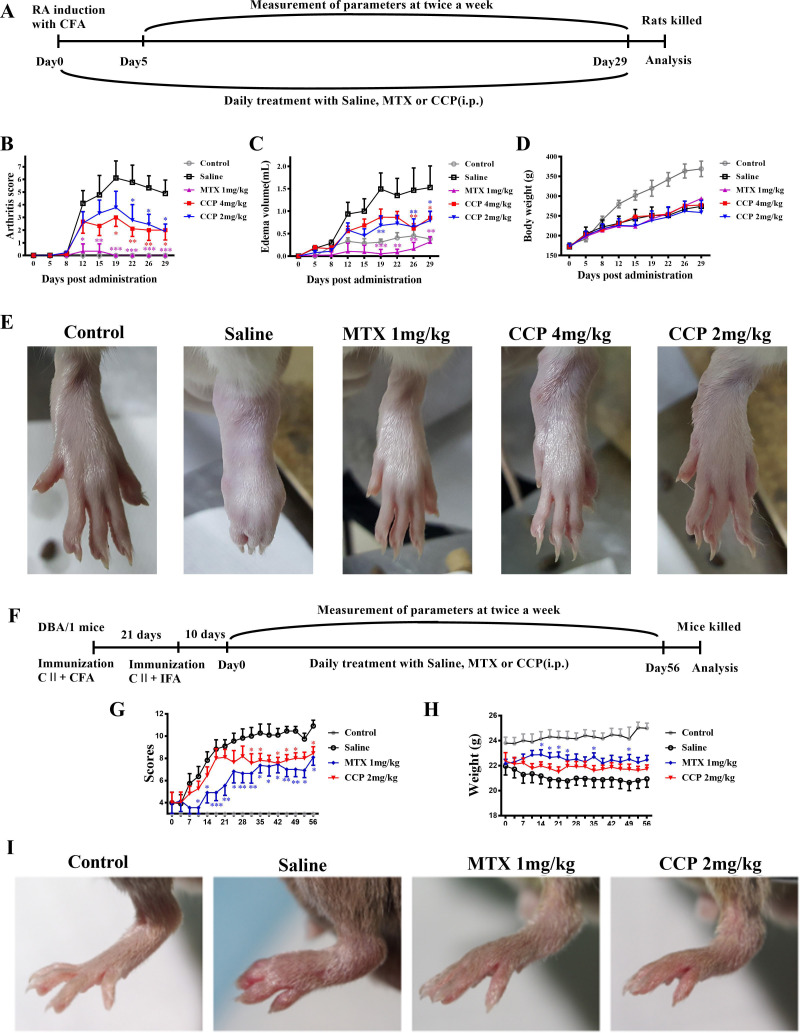
CCP treatment prevented arthritis progression in rodent arthritis. (A–E) Treatments and assessments of AIA rats: (A). Timetable of AIA induction and treatment strategy in CFA-injected SD rats. Arthritis scores (B), secondary paw swelling (C) and body weight (D) were recorded twice a week since day 0 (E). Representative photographs of showing the gross features of left hind paws at day 24 post-treatment. (F–I) Treatments and assessments of CIA mice: (F). Timetable of CIA induction and treatment strategy in CII-immunised DBA/1J mice. Arthritis scores (G) and body weight (H) were recorded twice a week since day 0 (I). Representative photographs of showing the gross features of left hind paws at day 28 post-treatment. Values are expressed as mean±SD (n≥6). *P<0.05, **P<0.01, ***P<0.001 represents significant differences compared with the saline-treated AIA rats or CIA mice, respectively. AIA, adjuvant-induced arthritis; CCP, cervus and cucumis polypeptide; CFA, complete Freund’s adjuvant; CIA, collagen-induced arthritis; MTX, methotrexate; RA, rheumatoid arthritis; SD, Sprague-Dawley.

The AIA rats were intraperitoneal injected with saline, MTX or CCP for consecutive 29 days (figure 1A). AIA rats developed severe swelling of the left hind paws. In contrast, administration of MTX and CCP significantly blocked the progression of arthritis development (figure 1B,C), nevertheless, neither application of MTX nor of CCP could restore the body weight loss caused by the severe systemic inflammation in AIA rats (figure 1D). Representative photograph displaying morphological changes of left hind paws of rats from individual group, macroscopic evidence of arthritis such as erythema or swelling was markedly observed in saline-treated rats compared with controls, while MTX and CCP treatment significantly ameliorated arthritis severity in AIA rats (figure 1E).

The CIA mice were administrated intraperitoneally with saline, MTX or CCP for 56 days (figure 1F). As shown in figure 1G, administration of MTX and CCP significantly prevented the progression of arthritis development in CIA mice according to the mean arthritis scores. Similar as the results in AIA mice, neither application of MTX nor of CCP could restore the body weight loss in CIA mice (figure 1H).

Moreover, prime arthritis symptoms, such as swelling and erythema in CIA mice, was significant in paws of saline-treated mice, which was obviously relieved after MTX and CCP treatment, as shown in the representative photographs of the hind paws captured on day 28 (figure 1I).

In comparison with saline-treated mice, the levels of serum anti-CII total IgG, IgG_1_ and IgG_2a_ Abs reduced significantly in CCP-treated CIA mice. However, level of serum anti-CII IgG_3_ antibody was not varied after CCP treatment in CIA mice (online supplementary figure S1).

### Micro-CT scan and analysis

**Figure 2:**
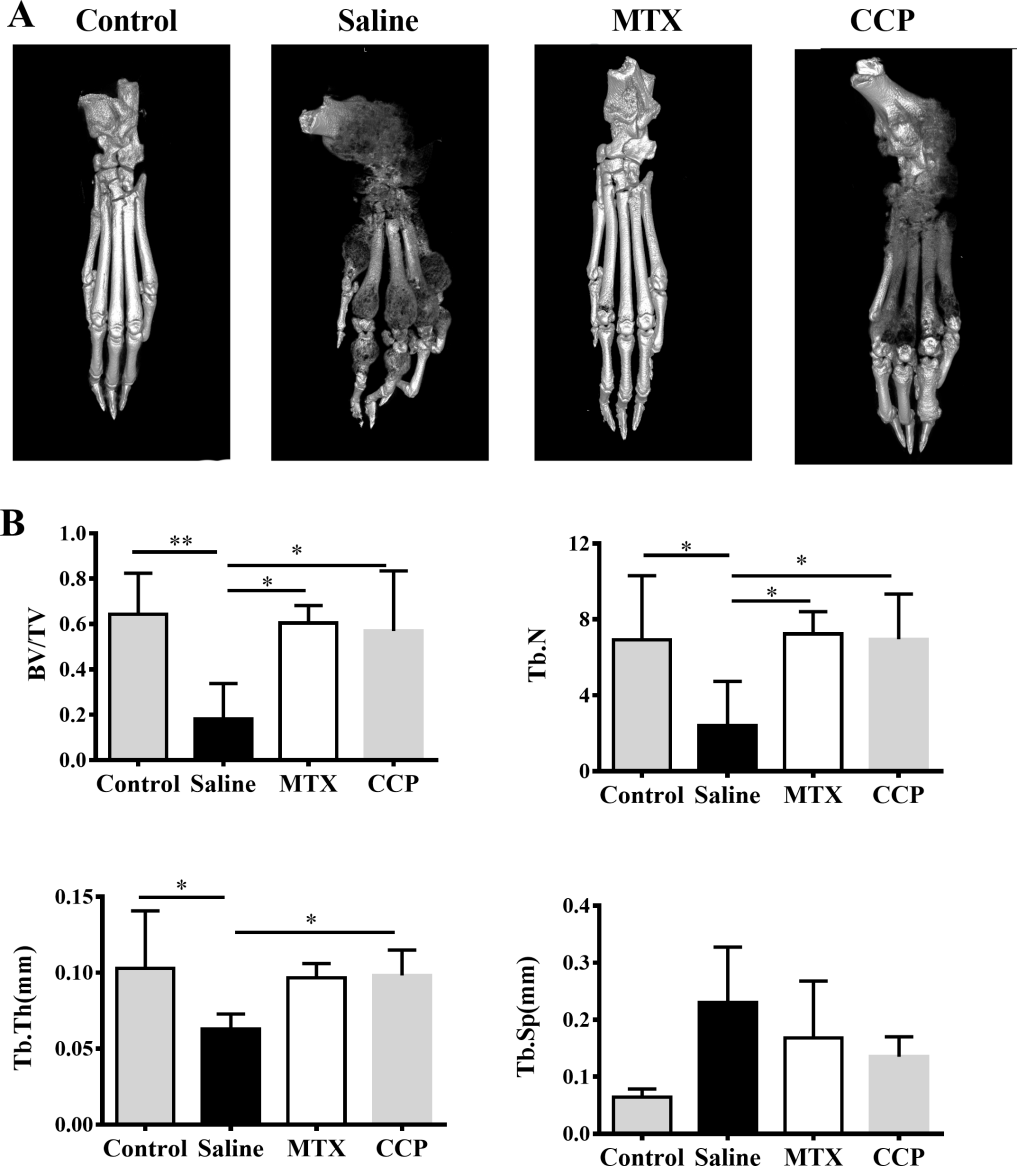
CCP treatment attenuated bone erosion in AIA rats. (A) Representative three-dimensional reconstruction of left hind paw of control mice and AIA rats treated with saline, MTX or CCP, respectively. (B) Histomorphometric analysis of the distal tibia for each treatment group, showing the values of four parameters including bone volume/total volume (BV/TV), trabecular number (Tb. N), trabecular thickness (Tb. Th.), and trabecular spacing (TB. Sp.) of control rats and AIA rats treated with saline or CCP. values are the mean±SD (n≥6). *P<0.05, **P<0.01 represent significant differences compared with the saline-treated AIA rats. AIA, adjuvant-induced arthritis; CCP, cervus and cucumis polypeptide; MTX, methotrexate.

Three-dimensional reconstruction of left hind paws by micro-CT demonstrated rough bone surface and severe bone erosion within their periarticular bone of paws of the saline-treated AIA rats, in marked contrast to normal SD rats, as well as to CCP-treated AIA rats (figure 2A). Moreover, as shown in figure 2B, saline-treated AIA rats showed a remarkable decrease of the bone volume/total volume (bone volume fraction, BV/TV), trabecular number (Tb. N.), and trabecular thickness (Tb. Th.), compared with controls. CCP treatment significantly prevented these changes in BV/TV, Tb. N. and Tb. Th. Although the trabecular spacing (Tb. Sp.) was higher in CCP-treated group compared with the saline-treated group, there was no statistically significant difference.

### CCP treatment attenuated bone destruction and reduced osteoclast numbers in AIA rats

**Figure 3:**
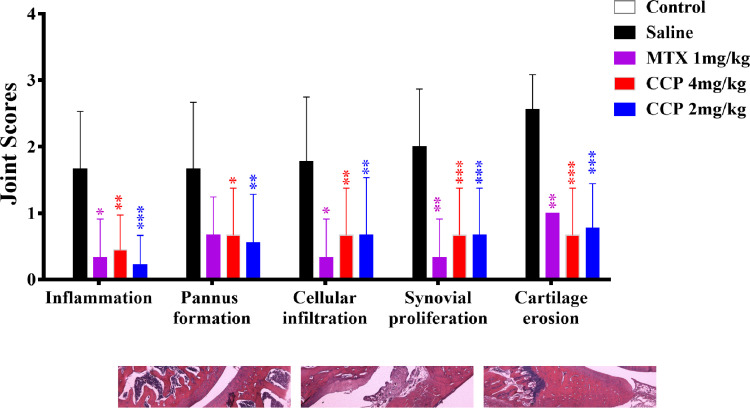
CCP treatment protected against the histological damage and reduced osteoclast numbers in AIA rats. The hind paws were prepared for histopathological examination on day 30 (A). Left panel: representative pathological sections of the ankle joint by H&E staining at the end of treatment; right panel: semiquantitative scores of histological examinations (B). Left panel: representative histochemical stains of the TRAP-positive cells in the joint tissues from rats of each group. Arrows indicated mature osteoclasts; right panel: numbers of TRAP-positive cells in field of whole joint section from rats of each group (n9). *P<0.05, **P<0.01, ***P<0.001 represent significant differences compared with the saline-treated AIA rats. AIA, adjuvant-induced arthritis; CCP, cervus and cucumis polypeptide; TRAP, tartrate resistant acid phosphatase.

In agreement with micro-CT analysis, the histological results show that the ankle joints in AIA rats exhibited apparent massive inflammatory cell infiltration in soft-tissue, synovial lining layer and cartilage destruction, as well as evident pannus formation and narrowed joint space. CCP or MTX treatment could restrict the histological development of AIA, as indicated by semiquantitative scores in pannus formation, cellular infiltration, proliferation of synoviocytes and joint tissues with resultant erosion of articular cartilage and bone (figure 3A).

To further explore whether osteoclasts were involved in the protective effects of CCP on AIA mice, we performed TRAP staining on tibia bone sections. Accordingly, TRAP staining revealed the significant decrease of TRAP-positive multinucleated cells at the trabecular surface and growth plates in CCP and MTX-treated AIA mice as compare with saline-treated AIA mice (figure 3B).

**Figure 4:**
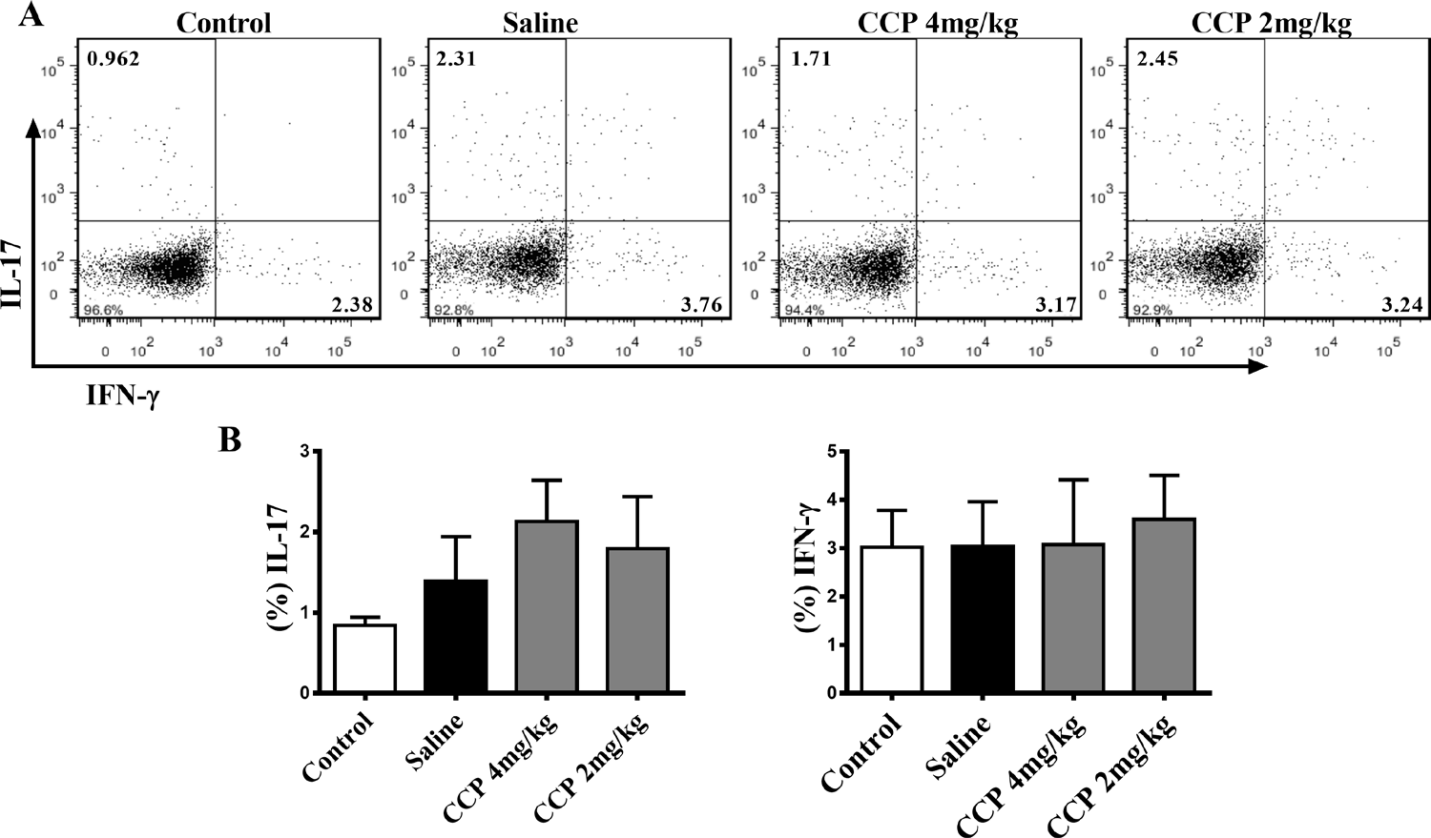
CCP treatment did not alter the splenic proportion of Th1 and Th17 cells in AIA rats. Splenocytes were isolated on day30 postadministration. The percentages of Th17 (CD3^+^CD4^+^IL-17^+^) and Th1 cells (CD3^+^CD4^+^IFN-γ^+^) were analysed by flow cytometry. Representative (A) and statistical (B) results were showed. Values are expressed as mean±SD (n=5). AIA, adjuvant-induced arthritis; CCP, cervus and cucumis polypeptide; Th1, type 1 T helper.

We also evaluated the impact of CCP on proinflammatory cytokines and immune cells, the essential participants in the pathogenesis of RA. Nevertheless, neither the serum IL-1β, IL-6, IL-17 or TNF-α (online supplementary figure S2), nor the splenic type 1 T helper (Th1) nor the Th17 proportion in AIA rats altered significantly after CCP treatment (figure 4). Similar results of CCP effects on pathogenic Th1 and Th17 cells were also obtained in the CIA mice (online supplementary data, Fig. S3).

Taken together, these data clearly indicate that CCP treatment could effectively reduce the joint damage and bone erosion without intervening proinflammatory cytokines expression and Th1/Th17 polarisation in rodent arthritis.

### CCP suppressed RANKL/M-CSF-induced osteoclast differentiation

**Figure 5:**
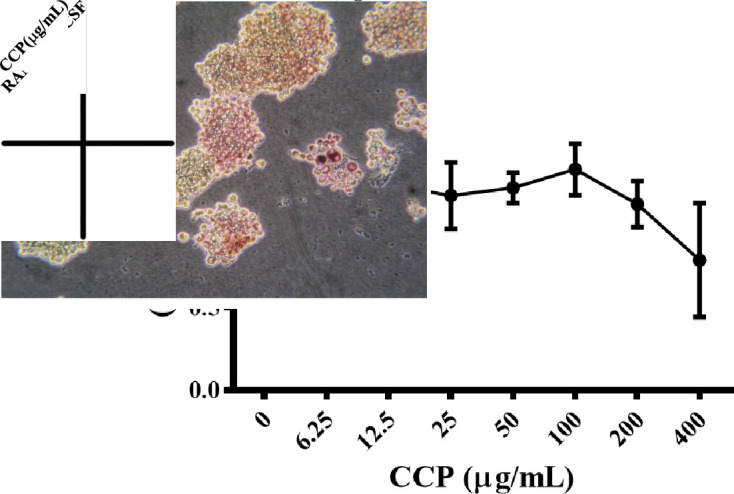
CCP suppressed RANKL/M-CSF-induced osteoclast differentiation. (A). CCK-8 assay was performed in triplicate to analyse the cell viability of RAW264.7 treated with indicated concentration of CCP for 4 days. (B). Representative trap staining images of RAW264.7 cells treated with or without CCP (100, 10 and 1 µg/mL), followed by RANKL/M-CSF stimulation for 4 days. (C) The number of TRAP-positive multinucleated osteoclasts (≥3 nuclei) was quantified. Data were expressed as means±SEM of independent experiments were performed. *P<0.05, **P<0.01 represent significant differences compared with cells stimulated by RANKL/M-CSF only. CCP, cervus and cucumis polypeptide; M-CSF, macrophage colony-stimulating factor; RANKL, receptor activator of nuclear factor-κB ligand; TRAP, tartrate resistant acid phosphatase.

On account of the protective effects of CCP on bone destruction in AIA rats, we, thus, further evaluated the direct role of CCP on osteoclastogenesis, in an in vitro culture system. At concentrations ranging from 6.25 to 100 µg/mL, CCP did not display any cellular toxicity against RAW264.7 cells (figure 5A).

Subsequently, RANKL/M-CSF-treated RAW264.7 cells were exposed to CCP for 4 days. RANKL/M-CSF stimulation induced the formation of osteoclast-like TRAP-positive multinuclear giant cells, and CCP significantly inhibited the osteoclast formation in a dose-dependent manner (figure 5B,C).

These data suggested that CCP is a potent inhibitor of osteoclastogenesis.

### CCP suppressed RANKL-induced downstream signalling activation

**Figure 6:**
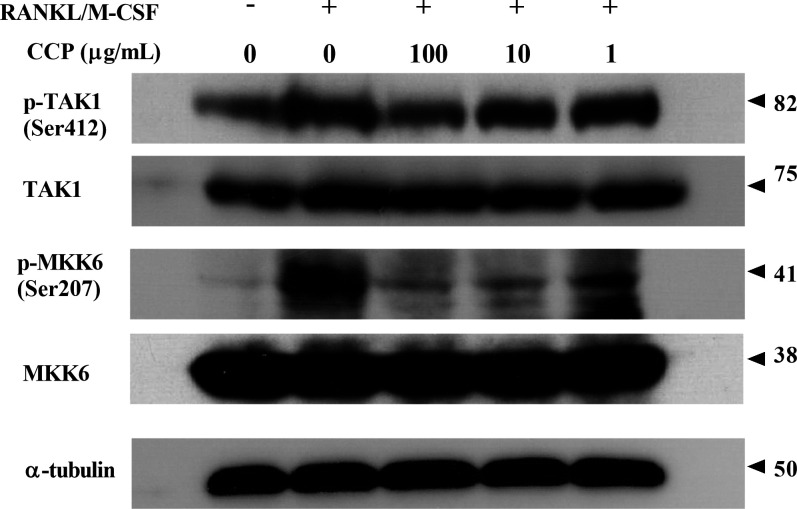
CCP suppressed RANKL-induced downstream signalling activation. RAW264.7 cells were treated with or without CCP (100, 10 and 1µg/mL), followed by treatment with RANKL/M-CSF for 72 hours. Cell lysates were subjected to western blot analysis. CCP, cervus and cucumis polypeptide; M-CSF, macrophage colony-stimulating factor; RANKL, receptor activator of nuclear factor-κB ligand.

RANKL-RANK binding recruits TRAFs to initiate the activation of downstream signalling cascades of adaptors/kinases. As shown in figure 6, the results indicated that CCP dose dependently attenuated the RANKL-induced phosphorylation of TAK1. Similarly, CCP showed inhibitory effect on the phosphorylation of MKK6 in response of RANKL stimulation (figure 6).

### CCP inhibited RANKL-induced osteoclast-specific genes and transcription factors expression in RAW264.7 cell

**Figure 7:**
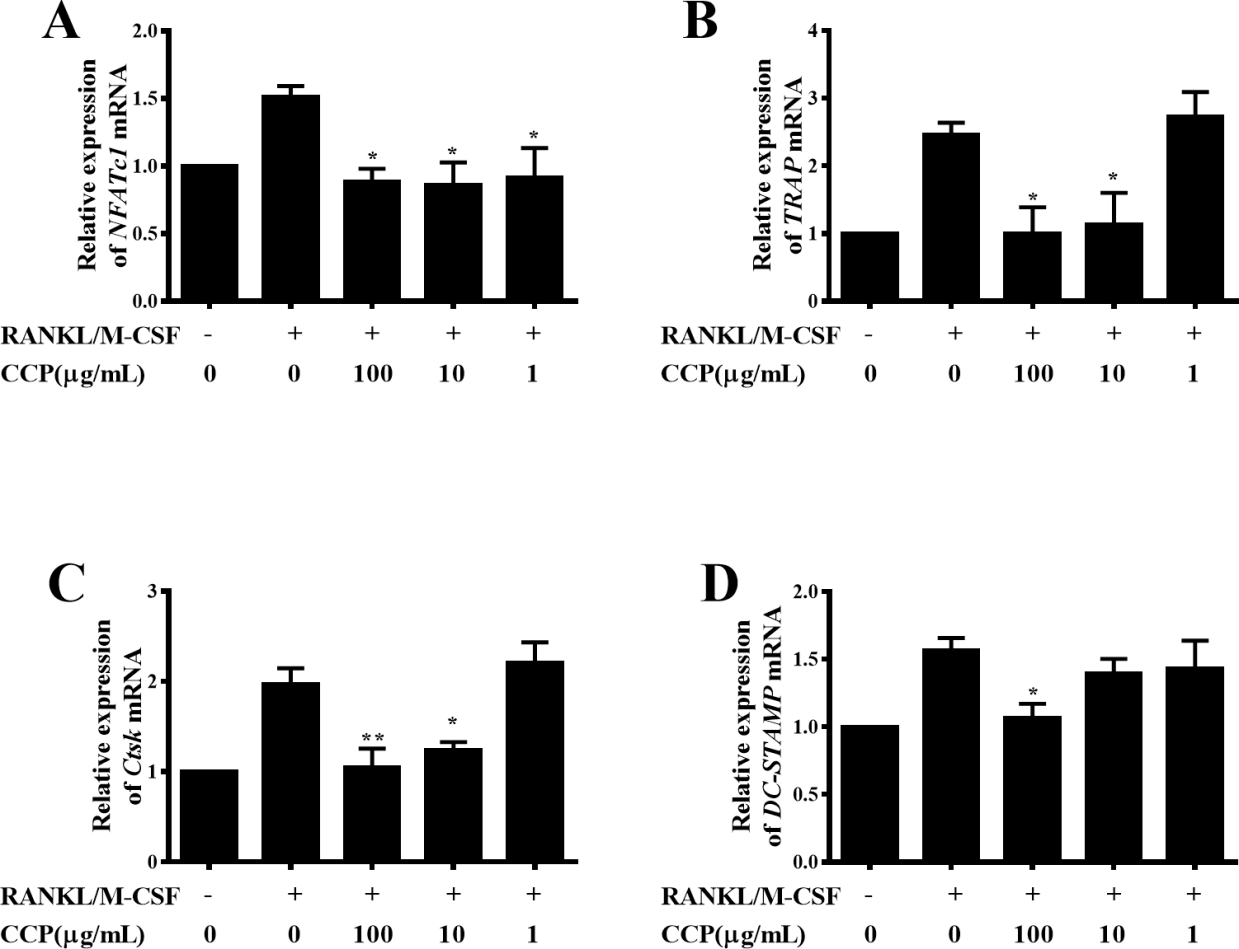
CCP inhibited RANKL-induced osteoclast-specific genes and transcription factors expression in RAW264.7 cell. RAW264.7 cells were pretreated with CCP (100, 10 and 1µg/mL) for 2 hours and then treated with RANKL/M-CSF for 24 hours. Real-time PCR was used to determine the expression of osteoclast marker gene NFATc1 (A), TRAP (B), Ctsk (C) and DC-STAMP (D). The messenger RNA (mRNA) expression was normalised to β-actin mRNA expression and converted to fold change of control. Data were expressed as means±SEM of independent experiments were performed. *P<0.05, **P<0.01 represent significant differences compared with cells stimulated by RANKL/M-CSF only. CCP, cervus and cucumis polypeptide; DC-STAMP, dendritic cell-specific transmembrane protein; M-CSF, macrophage colony-stimulating factor; RANKL, receptor activator of nuclear factor-κB ligand; TRAP, tartrate resistant acid phosphatase.

To further elucidate the molecular mechanisms of CCP-mediated inhibition of osteoclastogenesis, we investigated the effect of CCP on the expression of osteoclast-specific genes by real-time RT-PCR. As shown in figure 7A, addition of CCP significantly inhibited the increase of NFATc1 transcription induced by RANKL. Moreover, RANKL stimulation upregulated the levels of TRAP and Ctsk in RAW264.7 cells. In contrast, CCP treatment dose dependently inhibited the induction of these two genes (figure 7B,C).

Consistent with the expression of NFATc1, TRAP and Ctsk, stimulation with RANKL provoked the DC-STAMP expression in RAW264.7 cells, and the DC-STAMP level was markedly suppressed by CCP at 100 µg/mL (figure 7D).

Collectively, these results suggested that CCP restrained the osteoclast differentiation by suppressing the levels of osteoclast-specific genes and transcriptional factors, as well as the molecules mediated cell-cell fusion of osteoclasts.

## Discussion

The active ingredients of CCP injection are the combined extracts from deer horn and sweet melon seeds, which are used to promote fracture healing and treat osteoarthritis and RA in clinical practice.^16 27 28^ In the present study, daily treatment of AIA rats and CIA mice with CCP injection significantly ameliorated the paw oedema and bone destruction, as compared with saline treated ones. In addition, histological (H&E) and radiographical (micro-CT) evidence from ankle joints revealed that CCP treatment led to a remarkable improvement on bone erosion and joint damage. Further, in vitro supplementation of CCP dose dependently inhibited RANKL/M-CSF-induced osteoclast differentiation, suggested a direct impact of CCP on osteoclastogenesis, which might contribute to its protective effects against bone destruction in RA.

RA is a chronic inflammatory and osteodestructive process leads to an accumulation of synovial inflammation and joint damage over time. MTX is an antifolate metabolite that inhibits DNA synthesis, repair and cellular replication. It has anti-inflammatory properties and is used as an anchor disease-modifying anti-rheumatic drugs in treating RA.^29–31^ Although systemic inflammation plays a key role in a condition that profoundly affects the skeletal system, focal articular bone erosion is mainly mediated by osteoclasts.^16 32^ Although the repair of focal bone erosions has been accompanied with the control of inflammation, there are currently few therapeutic interventions have been identified to preventing bone destruction, the most severe outcome of this disease.^33 34^ Base on hospital-based observation, MTX benefits a large number of patients with RA but partially suffered from side effects. Thus, the CCP which performed less potency on RA animals than MTX, might be applied as a beneficial complement and alternative therapy for patients, who stopped taking MTX because of poor tolerance of side effects induced by it.

M-CSF and RANKL are two pivotal factors essential for osteoclast formation. During the early stage of osteoclast differentiation, M-CSF signalling induces osteoclasts proliferation and maintains their survival. RANKL induces NFATc1, AP-1 and NF-κB activation via TRAF6 recruitment and the MAPKs signalling cascades to promote their further differentiation into mature osteoclasts.^35^ In current study, we demonstrated that CCP treatment could prohibit the osteoclastogenesis in an RANKL/M-CSF-stimulated osteoclast precursors RAW264.7 culture in vitro (figure 5). Further, effect of CCP on osteoclastogenesis was associated with its suppression of RANKL-induced TAK1 and MKK6 phosphorylation, as well as the inhibition of osteoclastogenic genes including NFATc1, TRAP and Ctsk.

In addition, multinucleation is an essential step in the differentiation of osteoclasts. Osteoclast precursors fuse with one another and become multinucleated during maturation under the influence of RANKL. RANKL via NFATc1 induces the expression of fusion-mediating molecules such as DC-STAMP, by binding directly to their promoter regions.^36^ Consistent with the cellular morphological results, evidence in the mRNA expression of DC-STAMP in the RANKL-stimulated RAW264.7 cells suggested that CCP might interfere the fusion of macrophages through modulating the fusion-mediating molecules expression.

Although RANKL has been studied as a potential target for the treatment of RA, the precise mechanisms of osteoclast generation and activation in RA have not been fully elucidated. There are two receptors for RANKL, the membrane-bound signalling receptor RANK and the soluble decoy receptor osteoprotegerin (OPG). OPG blocks RANKL binding to RANK, thereby inhibiting osteoclastogenesis induced by RANKL.^37^ In the present study, we demonstrated that CCP exerted protective effects on AIA rats by preventing bone destruction directly, and the therapeutic benefits of CCP on RA were probably attributed to the restriction of osteoclastogenesis mediated by RANKL/RANK signalling cascade. Nevertheless, thorough investigation is needed to delineate the exact effect of CCP on the RANKL/RANK/OPG axis in RA, and to clarify whether CCP could regulate the expression of OPG on osteoblast or it could affect the cytokines milieu within the inflamed sites of bone erosion in arthritis.

In conclusion, our study showed that administration of CCP significantly inhibited the progression of arthritis both in the rat AIA and the mouse CIA models by ameliorated bone destruction and reduced osteoclast numbers. The therapeutic benefits of CCP could be attributed to its suppression on RANKL-induced osteoclastogenesis and expression of osteoclast-specific genes in osteoclast precursor cells.

## Data Availability

All data relevant to the study are included in the article or uploaded as supplementary information.
