# Correction: Cervus and cucumis peptides ameliorates bone erosion in experimental arthritis by inhibiting osteoclastogenesis

**DOI:** 10.1136/lupus-2019-000331corr1

**Published:** 2024-01-19

Lin Z, Liu Y, Xu Y, *et al*. Cervus and cucumis peptides ameliorates bone erosion in experimental arthritis by inhibiting osteoclastogenesis. *Lupus Sci Med* 2019;6:e000331. doi: 10.1136/lupus-2019-000331.

This article was previously published with an error.

Representative photographs of showing the gross features of left hind paws at day 28 post-treatment in figure 1I were provided incorrectly. The correct version is shown below. This does not affect the interpretation of data and conclusions.

**Figure 1:**
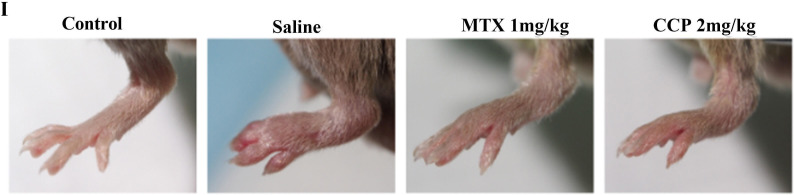
(I) Representative photographs of showing the gross features of left hind paws at day 28 post-treatment.

