# Supplementary material for: Cervus and cucumis peptides ameliorates bone erosion in experimental arthritis by inhibiting osteoclastogenesis
Source: Lupus Sci Med. 2019 May 11;6(1):e000331. doi: 10.1136/lupus-2019-000331 (PMC6519612; doi:10.1136/lupus-2019-000331)
Supplement: Supplementary data [file lupus-2019-000331supp001.pdf]

Cervus and Cucumis Peptides ameliorates bone erosion in experimental arthritis by inhibiting osteoclastogenesis

Zemin Lin[a, b, 1], Yuting Liu[a, c, 1], Yansheng Xu[a, b], Xiaoqian Yang[a], Fenghua Zhu[a], Wei Tang [a, c], Shijun He[a, c, *], Jianping Zuo[a, b, c,*]

[a] Laboratory of Immunopharmacology, State Key Laboratory of Drug Research, Shanghai Institute of Materia Medica, Chinese Academy of Sciences, Shanghai 201203, People's Republic of China

[b] Laboratory of Immunology and Virology, Shanghai University of Traditional Chinese Medicine, Shanghai 201203, People's Republic of China

[c] University of Chinese Academy of Sciences, No. 19A Yuquan Road, Beijing 100049, China

[*]Corresponding authors at: No .555 Zuchongzhi Road, Shanghai 201203, P. R. China.

Tel. & Fax: +86-21-50806701

E-mail addresses: jpzuo@simm.ac.cn (J. Zuo), heshijun@simm.ac.cn (S. He).

[1] These authors contributed equally to this work.

Supplementary Figure. S1

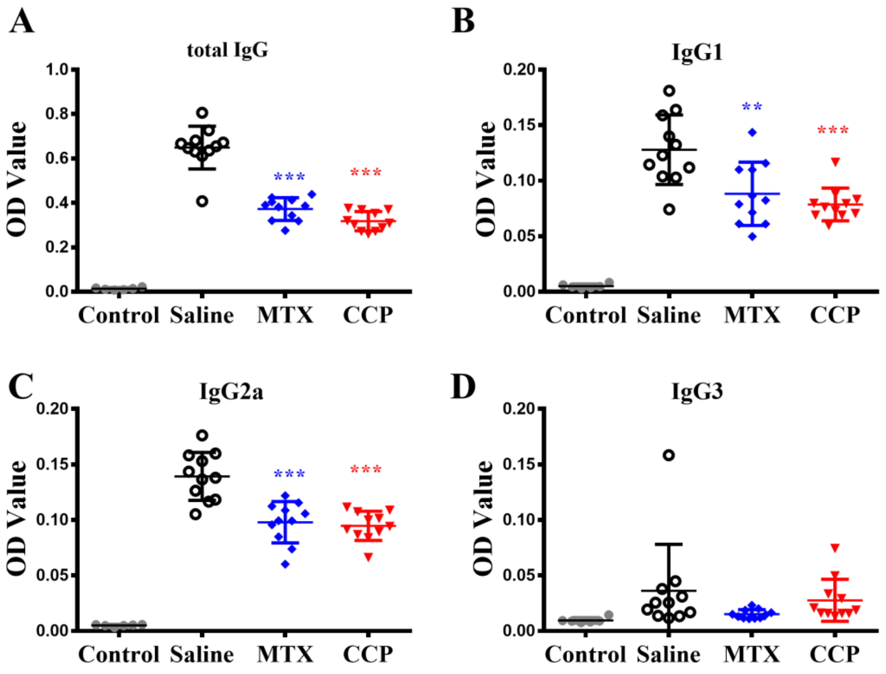

Fig. S1. Serum levels of CII-specific antibody in CIA mice. Serum (A) total anti-CII IgG, (B) IgG1, (C) IgG2a, and (D) IgG3 of CIA mice and control mice. Values are the mean ± SD ($n \geq 6$). **$P < 0.01$ and ***$P < 0.001$ compared with the saline-treated CIA mice.

Supplementary Figure. S2

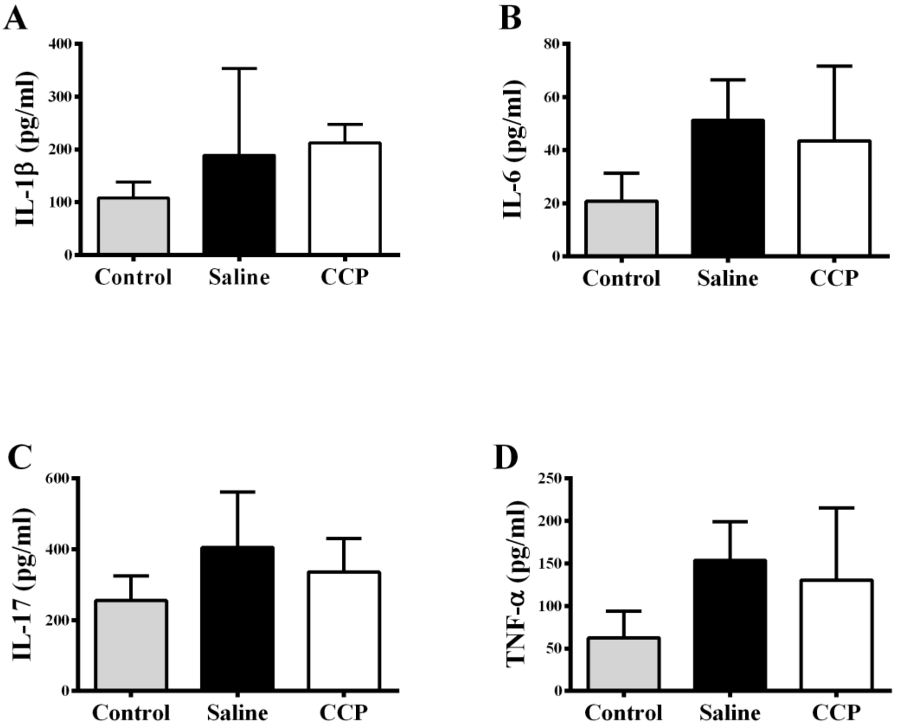

Fig. S2. Serum levels of inflammatory cytokines in AIA rats. At the termination of treatment, serum of AIA and control rats were collected and examined for cytokines. (A-D) Serum levels of IL-1β, IL-6, IL-17, and TNF-α. Values are the mean ± SD (n ≥ 6).

Supplementary Figure. S3

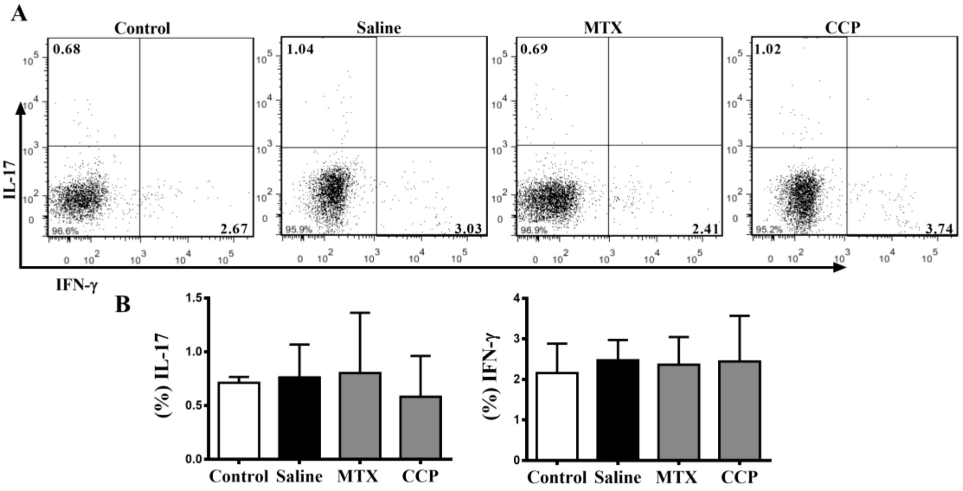

Fig. S3. Splenic proportion of Th1 and Th17 cells in CIA mice. Splenocytes were isolated on day 56 post administration. The percentage of Th17 ($CD3^+CD4^+IL\text{-}17^+$) and Th1 cells ($CD3^+CD4^+IFN\text{-}\gamma^+$) were analyzed by flow cytometry. Representative (A) and statistical (B) results were showed. Values are expressed as mean ± SD (n = 3).
